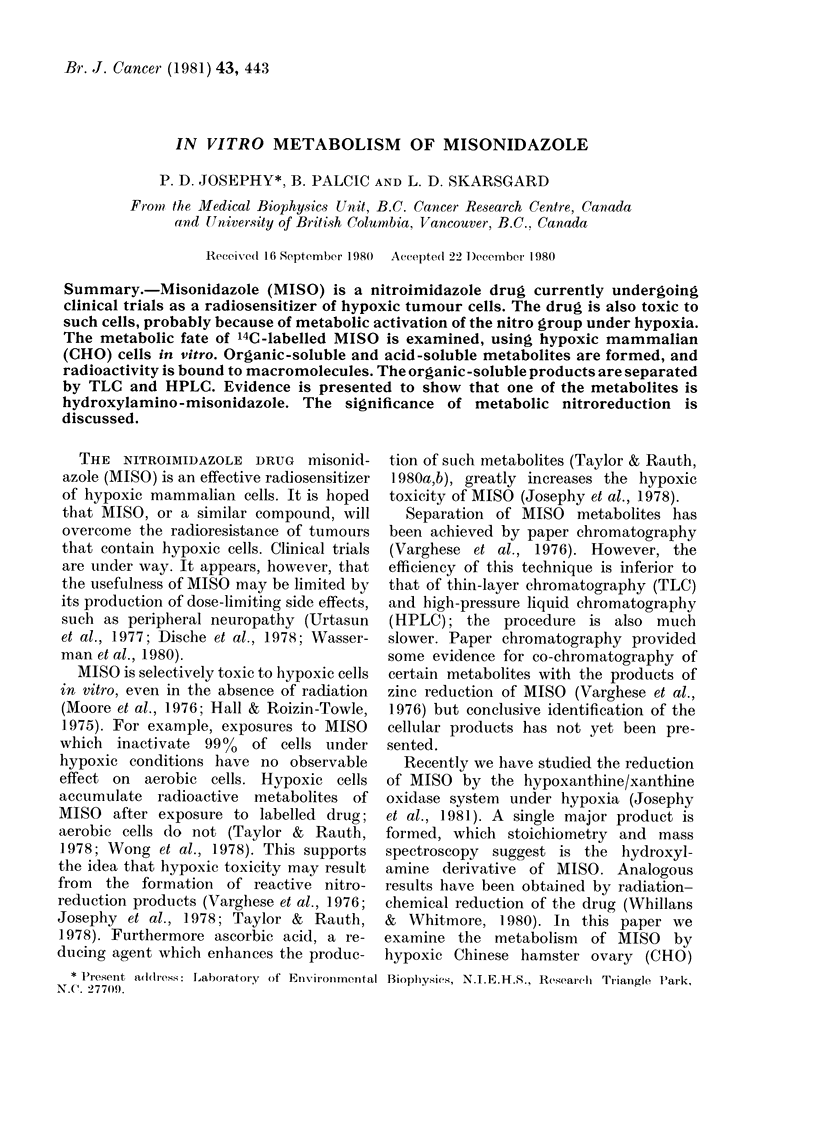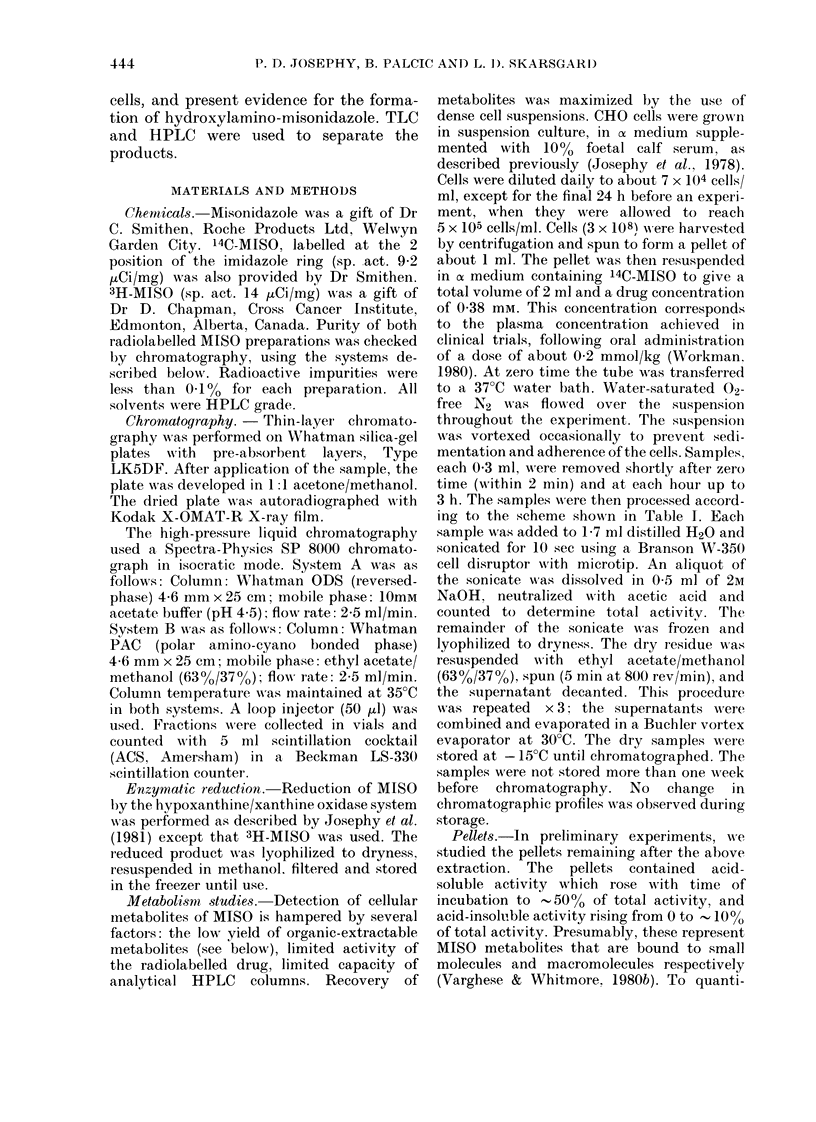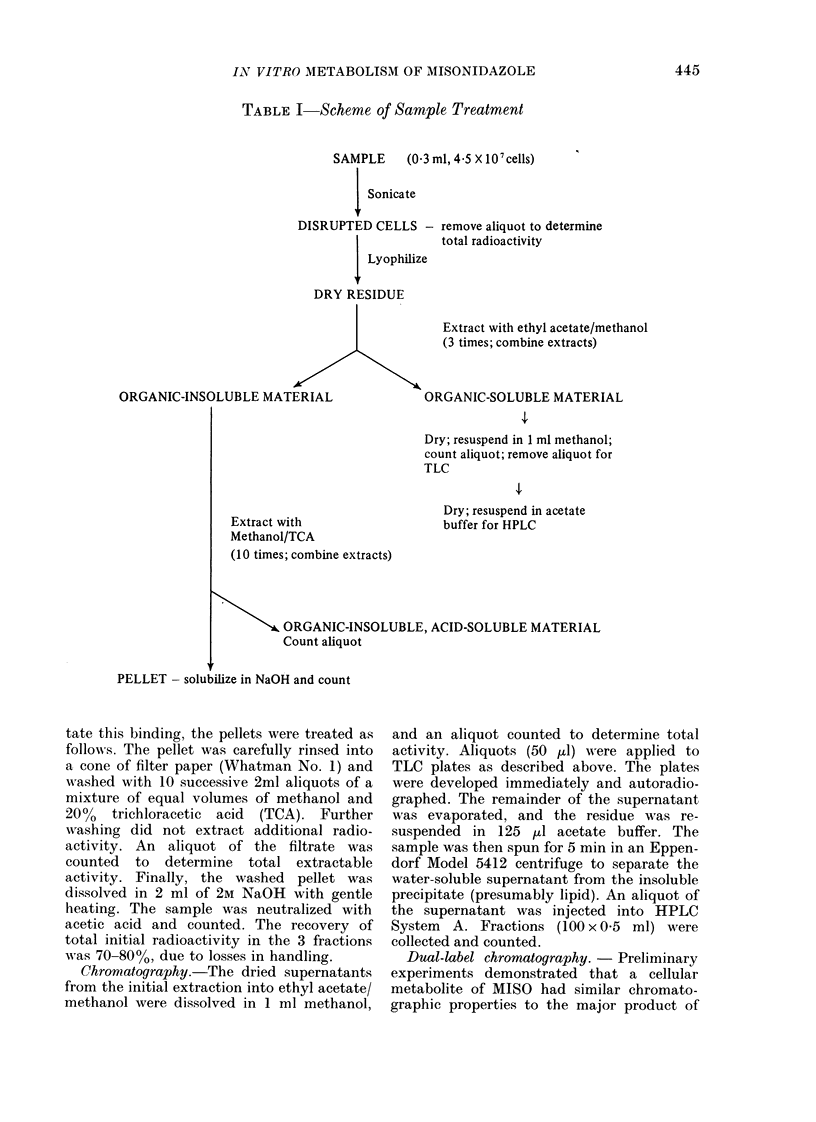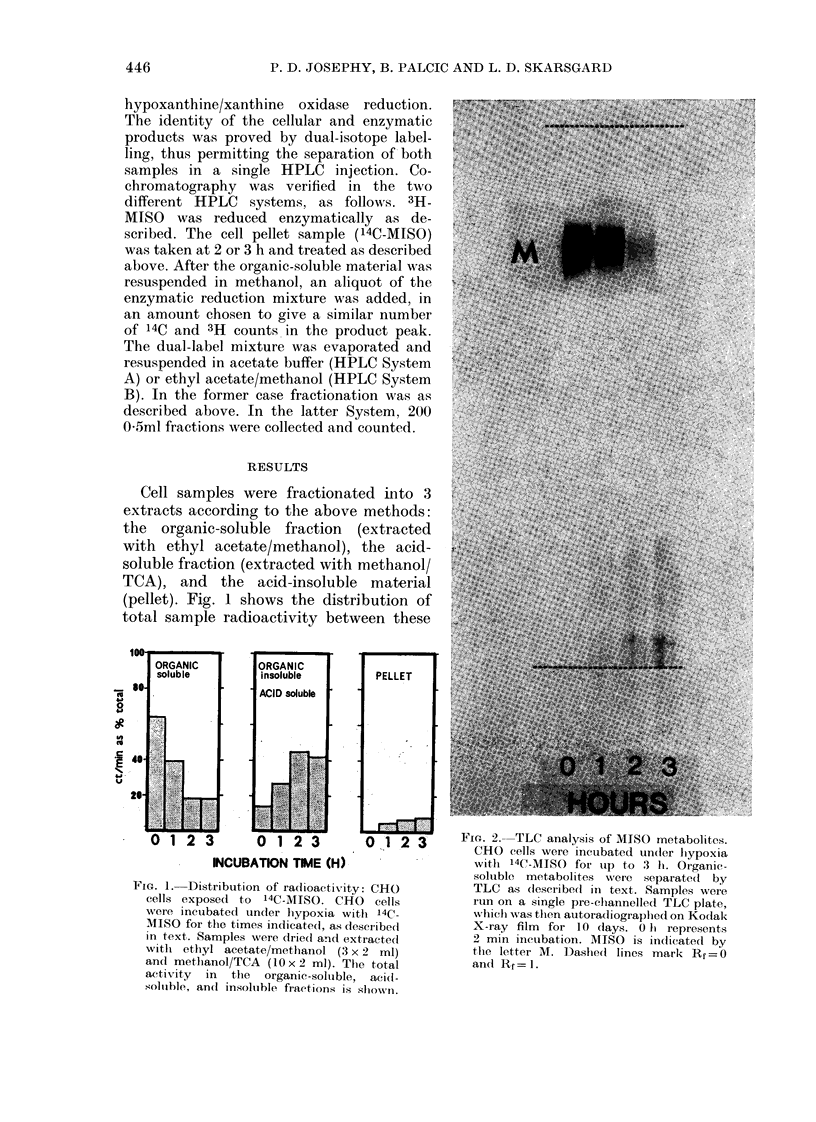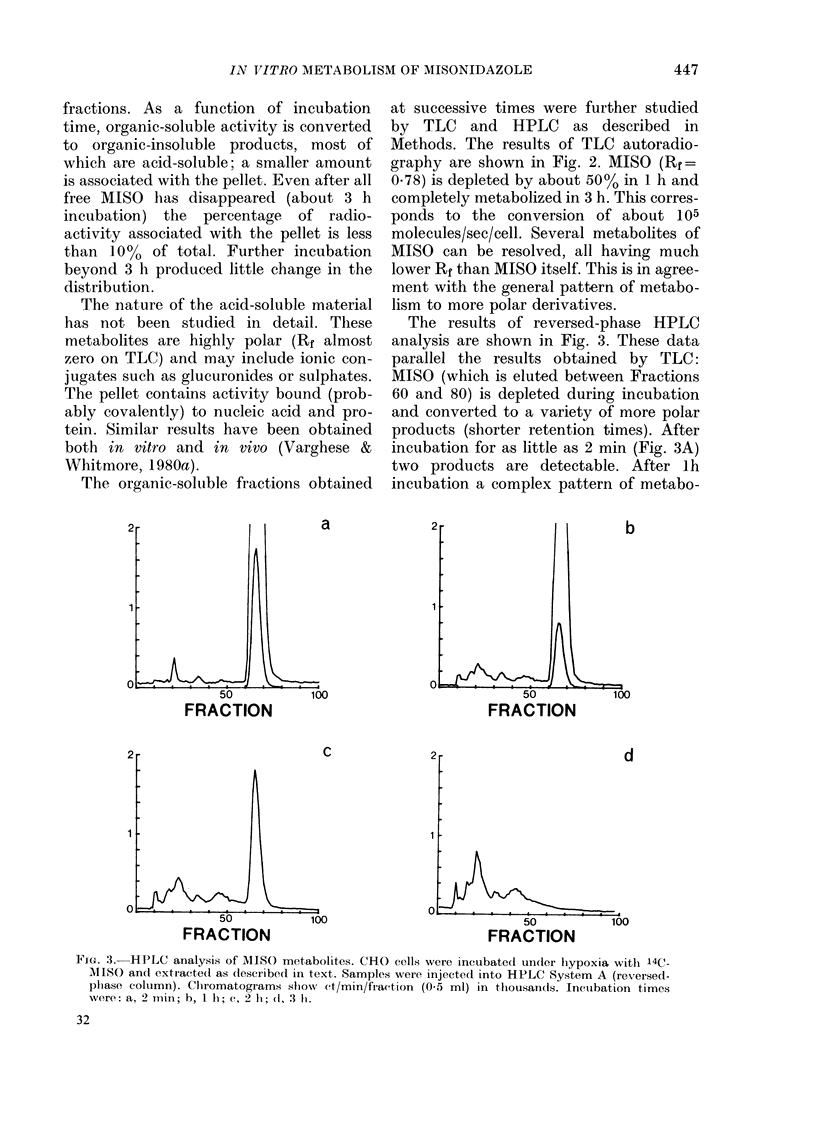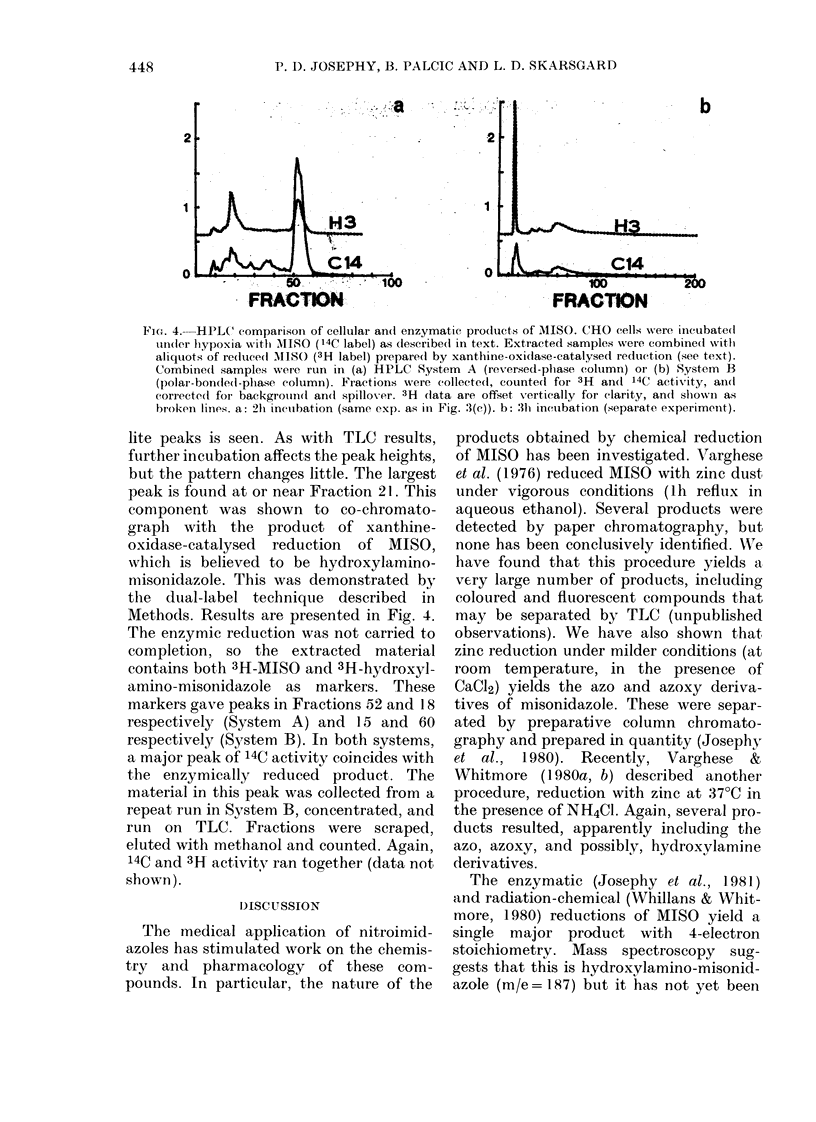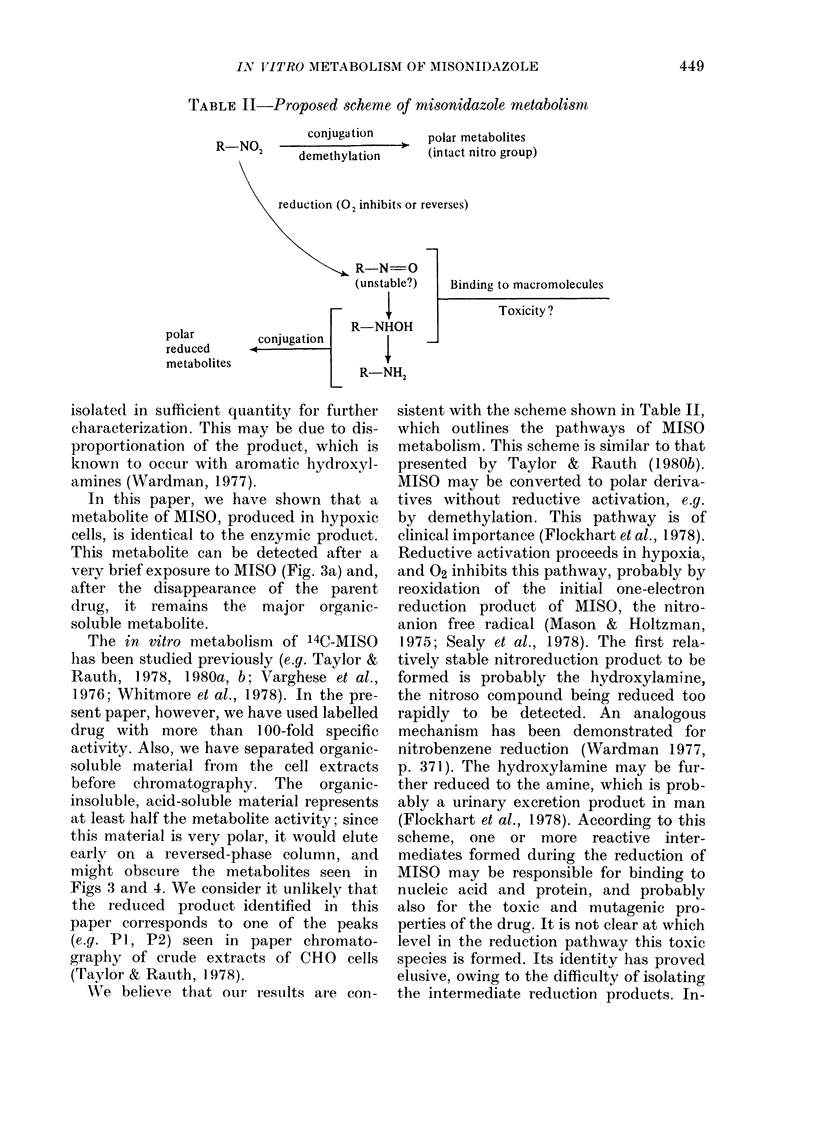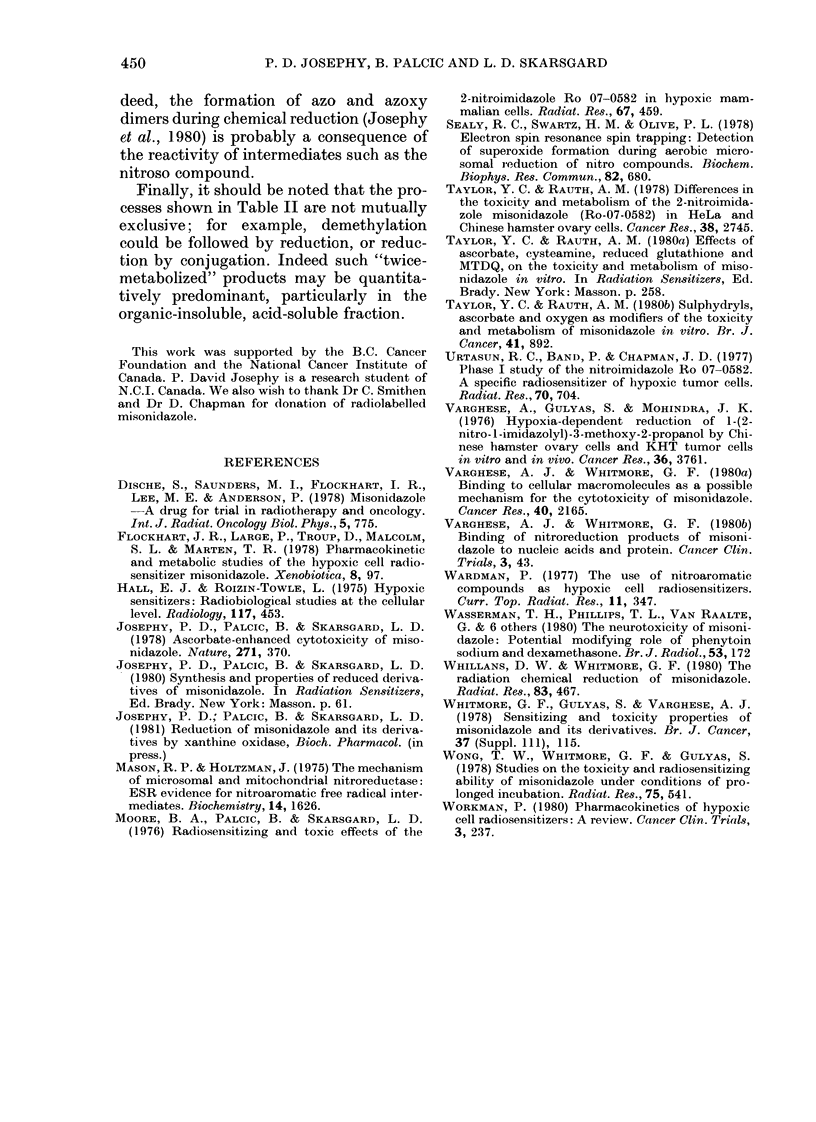# In vitro metabolism of misonidazole.

**DOI:** 10.1038/bjc.1981.65

**Published:** 1981-04

**Authors:** P. D. Josephy, B. Palcic, L. D. Skarsgard

## Abstract

**Images:**


*Br. J. Cancer* (1981) **43**, 443

### *IN VITRO* METABOLISM OF MISONIDAZOLE

P. D. JOSEPHY*, B. PALCIC AND L. D. SKARSGARD

*From the Medical Biophysics Unit, B.C. Cancer Research Centre, Canada and University of British Columbia, Vancouver, B.C., Canada*

Received 16 September 1980 Accepted 22 December 1980

**Summary.—Misonidazole (MISO) is a nitroimidazole drug currently undergoing clinical trials as a radiosensitizer of hypoxic tumour cells. The drug is also toxic to such cells, probably because of metabolic activation of the nitro group under hypoxia. The metabolic fate of $^{14}C$-labelled MISO is examined, using hypoxic mammalian (CHO) cells *in vitro*. Organic-soluble and acid-soluble metabolites are formed, and radioactivity is bound to macromolecules. The organic-soluble products are separated by TLC and HPLC. Evidence is presented to show that one of the metabolites is hydroxylamino-misonidazole. The significance of metabolic nitroreduction is discussed.**

THE NITROIMIDAZOLE DRUG misonidazole (MISO) is an effective radiosensitizer of hypoxic mammalian cells. It is hoped that MISO, or a similar compound, will overcome the radioresistance of tumours that contain hypoxic cells. Clinical trials are under way. It appears, however, that the usefulness of MISO may be limited by its production of dose-limiting side effects, such as peripheral neuropathy (Urtasun *et al.*, 1977; Dische *et al.*, 1978; Wasserman *et al.*, 1980).

MISO is selectively toxic to hypoxic cells *in vitro*, even in the absence of radiation (Moore *et al.*, 1976; Hall & Roizin-Towle, 1975). For example, exposures to MISO which inactivate 99% of cells under hypoxic conditions have no observable effect on aerobic cells. Hypoxic cells accumulate radioactive metabolites of MISO after exposure to labelled drug; aerobic cells do not (Taylor & Rauth, 1978; Wong *et al.*, 1978). This supports the idea that hypoxic toxicity may result from the formation of reactive nitroreduction products (Varghese *et al.*, 1976; Josephy *et al.*, 1978; Taylor & Rauth, 1978). Furthermore ascorbic acid, a reducing agent which enhances the production of such metabolites (Taylor & Rauth, 1980*a,b*), greatly increases the hypoxic toxicity of MISO (Josephy *et al.*, 1978).

Separation of MISO metabolites has been achieved by paper chromatography (Varghese *et al.*, 1976). However, the efficiency of this technique is inferior to that of thin-layer chromatography (TLC) and high-pressure liquid chromatography (HPLC); the procedure is also much slower. Paper chromatography provided some evidence for co-chromatography of certain metabolites with the products of zinc reduction of MISO (Varghese *et al.*, 1976) but conclusive identification of the cellular products has not yet been presented.

Recently we have studied the reduction of MISO by the hypoxanthine/xanthine oxidase system under hypoxia (Josephy *et al.*, 1981). A single major product is formed, which stoichiometry and mass spectroscopy suggest is the hydroxylamine derivative of MISO. Analogous results have been obtained by radiation–chemical reduction of the drug (Whillans & Whitmore, 1980). In this paper we examine the metabolism of MISO by hypoxic Chinese hamster ovary (CHO)

* Present address: Laboratory of Environmental Biophysics, N.I.E.H.S., Research Triangle Park, N.C. 27709.

cells, and present evidence for the formation of hydroxylamino-misonidazole. TLC and HPLC were used to separate the products.

### MATERIALS AND METHODS

*Chemicals.*—Misonidazole was a gift of Dr C. Smithen, Roche Products Ltd, Welwyn Garden City. $^{14}$C-MISO, labelled at the 2 position of the imidazole ring (sp. act. 9·2 $\mu$Ci/mg) was also provided by Dr Smithen. $^{3}$H-MISO (sp. act. 14 $\mu$Ci/mg) was a gift of Dr D. Chapman, Cross Cancer Institute, Edmonton, Alberta, Canada. Purity of both radiolabelled MISO preparations was checked by chromatography, using the systems described below. Radioactive impurities were less than 0·1% for each preparation. All solvents were HPLC grade.

*Chromatography.* — Thin-layer chromatography was performed on Whatman silica-gel plates with pre-absorbent layers, Type LK5DF. After application of the sample, the plate was developed in 1:1 acetone/methanol. The dried plate was autoradiographed with Kodak X-OMAT-R X-ray film.

The high-pressure liquid chromatography used a Spectra-Physics SP 8000 chromatograph in isocratic mode. System A was as follows: Column: Whatman ODS (reversed-phase) 4·6 mm × 25 cm; mobile phase: 10mM acetate buffer (pH 4·5); flow rate: 2·5 ml/min. System B was as follows: Column: Whatman PAC (polar amino-cyano bonded phase) 4·6 mm × 25 cm; mobile phase: ethyl acetate/methanol (63%/37%); flow rate: 2·5 ml/min. Column temperature was maintained at 35°C in both systems. A loop injector (50 $\mu$l) was used. Fractions were collected in vials and counted with 5 ml scintillation cocktail (ACS, Amersham) in a Beckman LS-330 scintillation counter.

*Enzymatic reduction.*—Reduction of MISO by the hypoxanthine/xanthine oxidase system was performed as described by Josephy *et al.* (1981) except that $^{3}$H-MISO was used. The reduced product was lyophilized to dryness, resuspended in methanol, filtered and stored in the freezer until use.

*Metabolism studies.*—Detection of cellular metabolites of MISO is hampered by several factors: the low yield of organic-extractable metabolites (see below), limited activity of the radiolabelled drug, limited capacity of analytical HPLC columns. Recovery of metabolites was maximized by the use of dense cell suspensions. CHO cells were grown in suspension culture, in $\alpha$ medium supplemented with 10% foetal calf serum, as described previously (Josephy *et al.*, 1978). Cells were diluted daily to about $7 \times 10^4$ cells/ml, except for the final 24 h before an experiment, when they were allowed to reach $5 \times 10^5$ cells/ml. Cells ($3 \times 10^8$) were harvested by centrifugation and spun to form a pellet of about 1 ml. The pellet was then resuspended in $\alpha$ medium containing $^{14}$C-MISO to give a total volume of 2 ml and a drug concentration of 0·38 mM. This concentration corresponds to the plasma concentration achieved in clinical trials, following oral administration of a dose of about 0·2 mmol/kg (Workman, 1980). At zero time the tube was transferred to a 37°C water bath. Water-saturated $O_2$-free $N_2$ was flowed over the suspension throughout the experiment. The suspension was vortexed occasionally to prevent sedimentation and adherence of the cells. Samples, each 0·3 ml, were removed shortly after zero time (within 2 min) and at each hour up to 3 h. The samples were then processed according to the scheme shown in Table I. Each sample was added to 1·7 ml distilled $H_2O$ and sonicated for 10 sec using a Branson W-350 cell disruptor with microtip. An aliquot of the sonicate was dissolved in 0·5 ml of 2M NaOH, neutralized with acetic acid and counted to determine total activity. The remainder of the sonicate was frozen and lyophilized to dryness. The dry residue was resuspended with ethyl acetate/methanol (63%/37%), spun (5 min at 800 rev/min), and the supernatant decanted. This procedure was repeated ×3; the supernatants were combined and evaporated in a Buchler vortex evaporator at 30°C. The dry samples were stored at −15°C until chromatographed. The samples were not stored more than one week before chromatography. No change in chromatographic profiles was observed during storage.

*Pellets.*—In preliminary experiments, we studied the pellets remaining after the above extraction. The pellets contained acid-soluble activity which rose with time of incubation to ~50% of total activity, and acid-insoluble activity rising from 0 to ~10% of total activity. Presumably, these represent MISO metabolites that are bound to small molecules and macromolecules respectively (Varghese & Whitmore, 1980*b*). To quanti-

TABLE I—*Scheme of Sample Treatment*

tate this binding, the pellets were treated as follows. The pellet was carefully rinsed into a cone of filter paper (Whatman No. 1) and washed with 10 successive 2ml aliquots of a mixture of equal volumes of methanol and 20% trichloracetic acid (TCA). Further washing did not extract additional radioactivity. An aliquot of the filtrate was counted to determine total extractable activity. Finally, the washed pellet was dissolved in 2 ml of 2M NaOH with gentle heating. The sample was neutralized with acetic acid and counted. The recovery of total initial radioactivity in the 3 fractions was 70–80%, due to losses in handling.

*Chromatography.*—The dried supernatants from the initial extraction into ethyl acetate/methanol were dissolved in 1 ml methanol, and an aliquot counted to determine total activity. Aliquots (50 μl) were applied to TLC plates as described above. The plates were developed immediately and autoradiographed. The remainder of the supernatant was evaporated, and the residue was resuspended in 125 μl acetate buffer. The sample was then spun for 5 min in an Eppendorf Model 5412 centrifuge to separate the water-soluble supernatant from the insoluble precipitate (presumably lipid). An aliquot of the supernatant was injected into HPLC System A. Fractions (100 × 0·5 ml) were collected and counted.

*Dual-label chromatography.* — Preliminary experiments demonstrated that a cellular metabolite of MISO had similar chromatographic properties to the major product of hypoxanthine/xanthine oxidase reduction. The identity of the cellular and enzymatic products was proved by dual-isotope labelling, thus permitting the separation of both samples in a single HPLC injection. Co-chromatography was verified in the two different HPLC systems, as follows. $^{3}H$-MISO was reduced enzymatically as described. The cell pellet sample ($^{14}C$-MISO) was taken at 2 or 3 h and treated as described above. After the organic-soluble material was resuspended in methanol, an aliquot of the enzymatic reduction mixture was added, in an amount chosen to give a similar number of $^{14}C$ and $^{3}H$ counts in the product peak. The dual-label mixture was evaporated and resuspended in acetate buffer (HPLC System A) or ethyl acetate/methanol (HPLC System B). In the former case fractionation was as described above. In the latter System, 200 0·5ml fractions were collected and counted.

### RESULTS

Cell samples were fractionated into 3 extracts according to the above methods: the organic-soluble fraction (extracted with ethyl acetate/methanol), the acid-soluble fraction (extracted with methanol/TCA), and the acid-insoluble material (pellet). Fig. 1 shows the distribution of total sample radioactivity between these

Fig. 1.—Distribution of radioactivity: CHO cells exposed to $^{14}C$-MISO. CHO cells were incubated under hypoxia with $^{14}C$-MISO for the times indicated, as described in text. Samples were dried and extracted with ethyl acetate/methanol (3 × 2 ml) and methanol/TCA (10 × 2 ml). The total activity in the organic-soluble, acid-soluble, and insoluble fractions is shown.

Fig. 2.—TLC analysis of MISO metabolites. CHO cells were incubated under hypoxia with $^{14}C$-MISO for up to 3 h. Organic-soluble metabolites were separated by TLC as described in text. Samples were run on a single pre-channelled TLC plate, which was then autoradiographed on Kodak X-ray film for 10 days. 0 h represents 2 min incubation. MISO is indicated by the letter M. Dashed lines mark $R_f = 0$ and $R_f = 1$.

fractions. As a function of incubation time, organic-soluble activity is converted to organic-insoluble products, most of which are acid-soluble; a smaller amount is associated with the pellet. Even after all free MISO has disappeared (about 3 h incubation) the percentage of radioactivity associated with the pellet is less than 10% of total. Further incubation beyond 3 h produced little change in the distribution.

The nature of the acid-soluble material has not been studied in detail. These metabolites are highly polar ($R_f$ almost zero on TLC) and may include ionic conjugates such as glucuronides or sulphates. The pellet contains activity bound (probably covalently) to nucleic acid and protein. Similar results have been obtained both *in vitro* and *in vivo* (Varghese & Whitmore, 1980*a*).

The organic-soluble fractions obtained at successive times were further studied by TLC and HPLC as described in Methods. The results of TLC autoradiography are shown in Fig. 2. MISO ($R_f$ = 0·78) is depleted by about 50% in 1 h and completely metabolized in 3 h. This corresponds to the conversion of about $10^5$ molecules/sec/cell. Several metabolites of MISO can be resolved, all having much lower $R_f$ than MISO itself. This is in agreement with the general pattern of metabolism to more polar derivatives.

The results of reversed-phase HPLC analysis are shown in Fig. 3. These data parallel the results obtained by TLC: MISO (which is eluted between Fractions 60 and 80) is depleted during incubation and converted to a variety of more polar products (shorter retention times). After incubation for as little as 2 min (Fig. 3A) two products are detectable. After 1h incubation a complex pattern of metabo-

FIG. 3.—HPLC analysis of MISO metabolites. CHO cells were incubated under hypoxia with $^{14}$C-MISO and extracted as described in text. Samples were injected into HPLC System A (reversed-phase column). Chromatograms show ct/min/fraction (0·5 ml) in thousands. Incubation times were: a, 2 min; b, 1 h; c, 2 h; d, 3 h.

FIG. 4.—HPLC comparison of cellular and enzymatic products of MISO. CHO cells were incubated under hypoxia with MISO ($^{14}C$ label) as described in text. Extracted samples were combined with aliquots of reduced MISO ($^{3}H$ label) prepared by xanthine-oxidase-catalysed reduction (see text). Combined samples were run in (a) HPLC System A (reversed-phase column) or (b) System B (polar-bonded-phase column). Fractions were collected, counted for $^{3}H$ and $^{14}C$ activity, and corrected for background and spillover. $^{3}H$ data are offset vertically for clarity, and shown as broken lines. a: 2h incubation (same exp. as in Fig. 3(c)). b: 3h incubation (separate experiment).

lite peaks is seen. As with TLC results, further incubation affects the peak heights, but the pattern changes little. The largest peak is found at or near Fraction 21. This component was shown to co-chromatograph with the product of xanthine-oxidase-catalysed reduction of MISO, which is believed to be hydroxylamino-misonidazole. This was demonstrated by the dual-label technique described in Methods. Results are presented in Fig. 4. The enzymic reduction was not carried to completion, so the extracted material contains both $^{3}H$-MISO and $^{3}H$-hydroxylamino-misonidazole as markers. These markers gave peaks in Fractions 52 and 18 respectively (System A) and 15 and 60 respectively (System B). In both systems, a major peak of $^{14}C$ activity coincides with the enzymically reduced product. The material in this peak was collected from a repeat run in System B, concentrated, and run on TLC. Fractions were scraped, eluted with methanol and counted. Again, $^{14}C$ and $^{3}H$ activity ran together (data not shown).

### DISCUSSION

The medical application of nitroimidazoles has stimulated work on the chemistry and pharmacology of these compounds. In particular, the nature of the products obtained by chemical reduction of MISO has been investigated. Varghese *et al.* (1976) reduced MISO with zinc dust under vigorous conditions (1h reflux in aqueous ethanol). Several products were detected by paper chromatography, but none has been conclusively identified. We have found that this procedure yields a very large number of products, including coloured and fluorescent compounds that may be separated by TLC (unpublished observations). We have also shown that zinc reduction under milder conditions (at room temperature, in the presence of $CaCl_2$) yields the azo and azoxy derivatives of misonidazole. These were separated by preparative column chromatography and prepared in quantity (Josephy *et al.*, 1980). Recently, Varghese & Whitmore (1980*a*, *b*) described another procedure, reduction with zinc at 37°C in the presence of $NH_4Cl$. Again, several products resulted, apparently including the azo, azoxy, and possibly, hydroxylamine derivatives.

The enzymatic (Josephy *et al.*, 1981) and radiation-chemical (Whillans & Whitmore, 1980) reductions of MISO yield a single major product with 4-electron stoichiometry. Mass spectroscopy suggests that this is hydroxylamino-misonidazole (m/e = 187) but it has not yet been

TABLE II—*Proposed scheme of misonidazole metabolism*

isolated in sufficient quantity for further characterization. This may be due to disproportionation of the product, which is known to occur with aromatic hydroxylamines (Wardman, 1977).

In this paper, we have shown that a metabolite of MISO, produced in hypoxic cells, is identical to the enzymic product. This metabolite can be detected after a very brief exposure to MISO (Fig. 3a) and, after the disappearance of the parent drug, it remains the major organic-soluble metabolite.

The *in vitro* metabolism of $^{14}$C-MISO has been studied previously (*e.g.* Taylor & Rauth, 1978, 1980*a*, *b*; Varghese *et al.*, 1976; Whitmore *et al.*, 1978). In the present paper, however, we have used labelled drug with more than 100-fold specific activity. Also, we have separated organic-soluble material from the cell extracts before chromatography. The organic-insoluble, acid-soluble material represents at least half the metabolite activity; since this material is very polar, it would elute early on a reversed-phase column, and might obscure the metabolites seen in Figs 3 and 4. We consider it unlikely that the reduced product identified in this paper corresponds to one of the peaks (*e.g.* P1, P2) seen in paper chromatography of crude extracts of CHO cells (Taylor & Rauth, 1978).

We believe that our results are consistent with the scheme shown in Table II, which outlines the pathways of MISO metabolism. This scheme is similar to that presented by Taylor & Rauth (1980*b*). MISO may be converted to polar derivatives without reductive activation, *e.g.* by demethylation. This pathway is of clinical importance (Flockhart *et al.*, 1978). Reductive activation proceeds in hypoxia, and $O_2$ inhibits this pathway, probably by reoxidation of the initial one-electron reduction product of MISO, the nitro-anion free radical (Mason & Holtzman, 1975; Sealy *et al.*, 1978). The first relatively stable nitroreduction product to be formed is probably the hydroxylamine, the nitroso compound being reduced too rapidly to be detected. An analogous mechanism has been demonstrated for nitrobenzene reduction (Wardman 1977, p. 371). The hydroxylamine may be further reduced to the amine, which is probably a urinary excretion product in man (Flockhart *et al.*, 1978). According to this scheme, one or more reactive intermediates formed during the reduction of MISO may be responsible for binding to nucleic acid and protein, and probably also for the toxic and mutagenic properties of the drug. It is not clear at which level in the reduction pathway this toxic species is formed. Its identity has proved elusive, owing to the difficulty of isolating the intermediate reduction products. Indeed, the formation of azo and azoxy dimers during chemical reduction (Josephy *et al.*, 1980) is probably a consequence of the reactivity of intermediates such as the nitroso compound.

Finally, it should be noted that the processes shown in Table II are not mutually exclusive; for example, demethylation could be followed by reduction, or reduction by conjugation. Indeed such "twice-metabolized" products may be quantitatively predominant, particularly in the organic-insoluble, acid-soluble fraction.

This work was supported by the B.C. Cancer Foundation and the National Cancer Institute of Canada. P. David Josephy is a research student of N.C.I. Canada. We also wish to thank Dr C. Smithen and Dr D. Chapman for donation of radiolabelled misonidazole.

### REFERENCES

DISCHE, S., SAUNDERS, M. I., FLOCKHART, I. R., LEE, M. E. & ANDERSON, P. (1978) Misonidazole—A drug for trial in radiotherapy and oncology. *Int. J. Radiat. Oncology Biol. Phys.*, **5**, 775.

FLOCKHART, J. R., LARGE, P., TROUP, D., MALCOLM, S. L. & MARTEN, T. R. (1978) Pharmacokinetic and metabolic studies of the hypoxic cell radiosensitizer misonidazole. *Xenobiotica*, **8**, 97.

HALL, E. J. & ROIZIN-TOWLE, L. (1975) Hypoxic sensitizers: Radiobiological studies at the cellular level. *Radiology*, **117**, 453.

JOSEPHY, P. D., PALCIC, B. & SKARSGARD, L. D. (1978) Ascorbate-enhanced cytotoxicity of misonidazole. *Nature*, **271**, 370.

JOSEPHY, P. D., PALCIC, B. & SKARSGARD, L. D. (1980) Synthesis and properties of reduced derivatives of misonidazole. In *Radiation Sensitizers*, Ed. Brady. New York: Masson. p. 61.

JOSEPHY, P. D.; PALCIC, B. & SKARSGARD, L. D. (1981) Reduction of misonidazole and its derivatives by xanthine oxidase, *Bioch. Pharmacol.* (in press.)

MASON, R. P. & HOLTZMAN, J. (1975) The mechanism of microsomal and mitochondrial nitroreductase: ESR evidence for nitroaromatic free radical intermediates. *Biochemistry*, **14**, 1626.

MOORE, B. A., PALCIC, B. & SKARSGARD, L. D. (1976) Radiosensitizing and toxic effects of the 2-nitroimidazole Ro 07-0582 in hypoxic mammalian cells. *Radiat. Res.*, **67**, 459.

SEALY, R. C., SWARTZ, H. M. & OLIVE, P. L. (1978) Electron spin resonance spin trapping: Detection of superoxide formation during aerobic microsomal reduction of nitro compounds. *Biochem. Biophys. Res. Commun.*, **82**, 680.

TAYLOR, Y. C. & RAUTH, A. M. (1978) Differences in the toxicity and metabolism of the 2-nitroimidazole misonidazole (Ro-07-0582) in HeLa and Chinese hamster ovary cells. *Cancer Res.*, **38**, 2745.

TAYLOR, Y. C. & RAUTH, A. M. (1980*a*) Effects of ascorbate, cysteamine, reduced glutathione and MTDQ, on the toxicity and metabolism of misonidazole *in vitro*. In *Radiation Sensitizers*, Ed. Brady. New York: Masson. p. 258.

TAYLOR, Y. C. & RAUTH, A. M. (1980*b*) Sulphydryls, ascorbate and oxygen as modifiers of the toxicity and metabolism of misonidazole *in vitro*. *Br. J. Cancer*, **41**, 892.

URTASUN, R. C., BAND, P. & CHAPMAN, J. D. (1977) Phase I study of the nitroimidazole Ro 07-0582. A specific radiosensitizer of hypoxic tumor cells. *Radiat. Res.*, **70**, 704.

VARGHESE, A., GULYAS, S. & MOHINDRA, J. K. (1976) Hypoxia-dependent reduction of 1-(2-nitro-1-imidazolyl)-3-methoxy-2-propanol by Chinese hamster ovary cells and KHT tumor cells *in vitro* and *in vivo*. *Cancer Res.*, **36**, 3761.

VARGHESE, A. J. & WHITMORE, G. F. (1980*a*) Binding to cellular macromolecules as a possible mechanism for the cytotoxicity of misonidazole. *Cancer Res.*, **40**, 2165.

VARGHESE, A. J. & WHITMORE, G. F. (1980*b*) Binding of nitroreduction products of misonidazole to nucleic acids and protein. *Cancer Clin. Trials*, **3**, 43.

WARDMAN, P. (1977) The use of nitroaromatic compounds as hypoxic cell radiosensitizers. *Curr. Top. Radiat. Res.*, **11**, 347.

WASSERMAN, T. H., PHILLIPS, T. L., VAN RAALTE, G. & 6 others (1980) The neurotoxicity of misonidazole: Potential modifying role of phenytoin sodium and dexamethasone. *Br. J. Radiol.*, **53**, 172

WHILLANS, D. W. & WHITMORE, G. F. (1980) The radiation chemical reduction of misonidazole. *Radiat. Res.*, **83**, 467.

WHITMORE, G. F., GULYAS, S. & VARGHESE, A. J. (1978) Sensitizing and toxicity properties of misonidazole and its derivatives. *Br. J. Cancer*, **37** (Suppl. 111), 115.

WONG, T. W., WHITMORE, G. F. & GULYAS, S. (1978) Studies on the toxicity and radiosensitizing ability of misonidazole under conditions of prolonged incubation. *Radiat. Res.*, **75**, 541.

WORKMAN, P. (1980) Pharmacokinetics of hypoxic cell radiosensitizers: A review. *Cancer Clin. Trials*, **3**, 237.